# Mass Spectrometric Behavior and Molecular Mechanisms of Fermented Deoxyanthocyanidins to Alleviate Ulcerative Colitis Based on Network Pharmacology

**DOI:** 10.1155/2022/9293208

**Published:** 2022-03-21

**Authors:** Yunpeng Bai, Guangwen Wang, Jinhua Lan, Ping Wu, Guowu Liang, Jinhui Huang, Zheng Wu, Yirong Wang, Chunbo Chen

**Affiliations:** ^1^Center of Scientific Research, Maoming People's Hospital, Maoming 525000, China; ^2^Department of Gastroenterology, Maoming People's Hospital, Maoming 525000, China; ^3^Department of Neurology, Maoming People's Hospital, Maoming 525000, China; ^4^Department of Critical Care Medicine, Guangdong Provincial People's Hospital, Guangdong Academy of Medical Sciences, Guangzhou 510080, China; ^5^School of Biology and Biological Engineering, South China University of Technology, Guangzhou 510006, China; ^6^Department of Critical Care Medicine, Maoming People's Hospital, Maoming 525000, China; ^7^Department of Emergency, Maoming People's Hospital, Maoming 525000, China

## Abstract

**Aims:**

Ulcerative colitis (UC) is a type of chronic idiopathic inflammatory bowel disease with a multifactorial pathogenesis and limited treatment options. The aim of the present study is to investigate the hydrogen deuterium exchange mass spectrometry (HDX-MS) behaviors of fermented deoxyanthocyanidins and their molecular mechanisms to alleviate UC by using quantum chemistry and network pharmacology.

**Methods:**

Tandem MS indicated at least two fragmentation pathways through which deuterated vinylphenol-deoxyanthocyanidins could generate different product ions. Quantum calculations were conducted to determine the transition states of the relevant molecules and analyze their optimized configuration, vibrational characteristics, intrinsic reaction coordinates, and corresponding energies. The potential targets of deoxyanthocyanidins in UC were screened from a public database. The *R* package was used for Gene Ontology (GO) and KEGG pathway analyses, and the protein–protein interactions (PPIs) of the targets were assessed using Search Tool for the Retrieval of Interacting Genes (STRING). Finally, molecular docking was implemented to analyze the binding energies and action modes of the target compounds through the online tool CB-Dock.

**Results:**

Quantum calculations indicated two potential fragmentation pathways involving the six-membered ring and dihydrogen cooperative transfer reactions of the vinylphenol-deoxyanthocyanidins. A total of 146 and 57 intersecting targets of natural and fermented deoxyanthocyanidins were separately screened out from the UC database and significant overlaps in GO terms and KEGG pathways were noted. Three shared hub targets (i.e., PTGS2, ESR1, and EGFR) were selected from the two PPI networks by STRING. Molecular docking results showed that all deoxyanthocyanidins have a good binding potential with the hub target proteins and that fermented deoxyanthocyanidins have lower binding energies and more stable conformations compared with natural ones.

**Conclusions:**

Deoxyanthocyanidins may provide anti-inflammatory, antioxidative, and immune system regulatory effects to suppress UC progression. It is proposed for the first time that fermentation of deoxyanthocyanidins can help adjust the structure of the intestinal microbiota and increase the biological activity of the natural compounds against UC. Furthermore, HDX-MS is a helpful strategy to analyze deoxyanthocyanidin metabolites with unknown structures.

## 1. Introduction

Ulcerative colitis (UC) is a chronic nonspecific inflammatory bowel disease (IBD) characterized by diffuse mucosal inflammation of the colon and rectum that may lead to permanent fibrosis, tissue damage, and a high risk of colitis-related colon cancer [1, 2]. The typical clinical features of UC include abdominal pain, diarrhea, mucopurulent bloody stools, a low cure rate, and easy recurrence [3]. The etiology and pathogenesis of UC have not been completely elucidated, and yet, the disease has become a highly refractory gastrointestinal condition with high incidence rates, increased risk of colon cancer, drug resistance, and adverse reactions [4]. Because no specific drugs are yet available for the disease, reasonable measures to alleviate the progress of UC, relieve its symptoms, and reduce recurrence rates are necessary [5].

The gut microbiota plays important roles in the cultivation of host immunity, digestion of food, regulation of intestinal endocrine function and neurological signaling, modification of drug action and metabolism, elimination of toxins, and production of numerous compounds influencing the host [6]. Evidence has revealed that the structures of the gut microbiota of IBD patients and healthy individuals show significant differences [7] and that alterations in the intestinal microflora markedly affect the severity and progression of colitis [8, 9]. Therefore, a new approach to UC treatment is the careful regulation of the intestinal microbiota [10].

Anthocyanins are a type of flavonoids commonly found in richly colored flowers and fruits [11]. Anthocyanins, like other polyphenols, have attracted wide attention on account of their strong antioxidative and anti-inflammatory capabilities, as well as their potential benefits to human health [12]. Several studies, such as an open pilot trial that sought to improve the symptoms of patients with mild to moderate UC [13], have reported that anthocyanin-rich extracts may help alleviate UC because of their excellent anti-inflammatory effects. Such extracts have also shown an effective ability to ameliorate disease severity in a dextran sodium sulfate (DSS)-induced rat colitis model [14]. 3-Deoxyanthocyanin is a relatively rare type of anthocyanin that mainly exists in sorghum; the compound is characterized by the absence of a hydroxyl group at position 3 of the flavylium ion [15]. Recent studies have shown that deoxyanthocyanins are more resistant than other anthocyanins to pH changes in the aqueous solution and more cytotoxic to cancer cells than their anthocyanindin analogs [16]. Thus, these rare pigments may have excellent potential applications as health-promoting phytochemicals.

**Figure 1:**
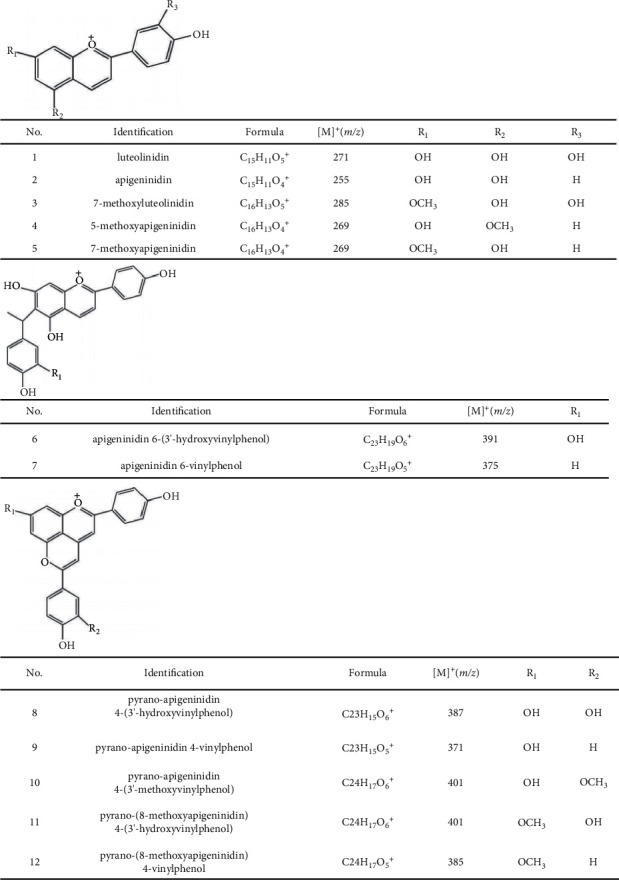
Chemical structures of 3-deoxyanthocyanidins, vinylphenol and pyrano adducts of fermented deoxyanthocyanidins. Modified from Literature 17 and 18.

In the previous study on sorghum deoxyanthocyanins fermented by *Lactobacillus plantarum*, novel vinylphenol and pyrano adducts of deoxyanthocyanins (Figure 1) were formed in sorghum sourdough [17, 18]. Hydrogen deuterium exchange mass spectrometry (HDX-MS) by using an LTQ-Orbitrap high-resolution MS (HRMS) system was then used to explore the fragmentation mechanisms of the fermented deoxyanthocyanins. MS technology is probably the most comprehensive and versatile analytical technique currently available and has various applications in chemical analysis, biochemistry, pharmaceutical sciences, and several other fields [19,20].

Network pharmacology could provide new methods to discover drugs to treat complex diseases and understand the multiple mechanisms of drug actions [21]. The construction of ‘disease-phenotype-gene-drug' networks by combining pharmacology, bioinformatics, and other sciences with systematic network analysis can help explain the mechanisms of multicomponent and multitarget drug treatment from the perspectives of gene distribution, molecular function, and signaling pathways [22, 23]. In the present research, network pharmacology was performed to elucidate the molecular targets and potential mechanisms of fermented deoxyanthocyanidins for further clinical and basic research on approaches to alleviate UC. Density functional theory (DFT) calculations were also carried out to investigate the MS fragmentation pathways of vinylphenol-deoxyanthocyanidins, which may be helpful in the analysis of the chemical structures of other unknown anthocyanin analogs.

## 2. Materials and Methods

### 2.1. HDX-MS Analysis and Quantum Chemistry Calculation

The detailed description of structural identification and the MS experiments for fermented deoxyanthocyanidins were published before [17]. Briefly, the HDX-MS experiments were performed as follows. Small amount of each purified compound was diluted by 1 mL of methanol/H_2_O (60 : 40, v/v) and directly injected into the LTQ-Orbitrap HRMS with ESI source at a flow rate of 15 *μ*L min^−1^. The HDX reactions were initiated by mixing each fermented deoxyanthocyanidin with 1 mL of precooled methanol-d4/D_2_O (60 : 40, v/v) to decrease H/*D* scrambling. The MS experiments included full scan and tandem MS in the positive ion mode for the fermented deoxyanthocyanidins and corresponding deuterated deoxyanthocyanidins. The ESI source was performed using the following parameters: spray voltage 4.5 kV; dry gas (N_2_) flow rate 15L min^−1^; capillary voltage 35V; capillary temperature 150 °C; and nebulizer (N_2_) pressure 35 psi. The higher energy collision-induced dissociation (HCD) mass spectra were obtained with a mass resolution of 17500 and normalized collision energy (NCE) from 15 to 50 eV.

DFT calculations were performed for geometry optimizations of deoxyanthocyanins by employing B3LYP/6-31G(d) hybrid functional implemented using the Gaussian 16 program [24]. Furthermore, we tried to verify the transitional states of compound 7 from the perspective of DFT calculations, including the geometrical structure optimization, vibration analysis, intrinsic reaction coordinate (IRC) method, and corresponding Gibbs free energies for the transition states. The relative Gibbs energies were calculated at 298 K and 1.0 atm for the comparison of fragmentation pathways.

### 2.2. Potential Targets of Deoxyanthocyanidins-UC Regulatory Network

The 2D structures of deoxyanthocyanidins were drawn through ChemDraw software, and the geometric configurations were optimized by Gaussian software. To obtain the targets of deoxyanthocyanidins, the SwissTargetPrediction database (http://www.swisstargetprediction.ch/) was used, which is a web server that predicts the most probable protein targets of small molecules based on the combination of 2D and 3D similarity measures of known ligands [25,26]. The SDF files of deoxyanthocyanidins were imported into the SwissTargetPrediction database for gene symbols of these identified candidate targets. To obtain the disease-related genes comprehensively, the UC-related genes were collected from GeneCards (https://www.genecards.org/) and DrugBank (https://www.drugbank.ca). Targets with hit scores greater than 9.0 were selected as the UC-related genes in the GeneCard database, and the UniProt database (http://www.uniprot.org/) was used for comparison of target information and gene symbol standardization. Subsequently, the regulatory networks of natural deoxyanthocyanidins-UC and fermented deoxyanthocyanidins-UC were generated by using Cytoscape software, respectively.

### 2.3. GO and KEGG Enrichment Analysis

Gene ontology (GO) analysis is a commonly used approach to define genes and their RNA or protein products to determine the unique biological characteristics of high-throughput transcriptome or genome data [27]. It is mainly used to study biological processes (BP), cellular composition (CC), and molecular function (MF). Kyoto Encyclopedia of Genes and Genomes (KEGG) is a collection of databases referring to genomes, diseases, biological pathways, drugs, and chemical materials [28]. In order to investigate the biological effects of deoxyanthocyanidins, GO and KEGG pathway enrichment analyses were conducted and calculated by the “clusterProfiler”, “enrichplot”, and “ggplot2” of *R* packages. The enriched GO terms and pathways with corrected *P* value less than 0.05 were selected and further analyzed.

### 2.4. Protein–Protein Interaction (PPI) Network and Topological Analysis

PPI is the basis of most biological processes in living cells and is essential for understanding cell physiology in normal and disease states. PPI network mappings originated from deoxyanthocyanidins and UC targets were performed using an online tool STRING (Search Tool for the Retrieval of Interacting Genes) (https://string-db.org/) with a confidence score ≥0.4 [29].

### 2.5. Molecular Docking

In order to investigate the relationship between the target proteins and ligands at the molecular level, molecular docking experiments were conducted to evaluate the interaction strength and mode between deoxyanthocyanidins and the targets. The docking simulation was performed by CB-Dock (http://cao.labshare.cn/cb-dock/), which can automatically recognize the binding sites of a given protein, calculate the center position, customize the docking box size, and use the popular docking program (AutoDock Vina) to dock with the query ligands [30]. 3D crystal structures of the target proteins were downloaded from the protein data bank (PDB) (http://www.rcsb.org), and the selected deoxyanthocyanidins were optimized by Gaussian software. Then, the protein and the deoxyanthocyanidins were uploaded to CB-Dock for docking. The docking scores were used to compare the theoretical binding affinities of natural and fermented deoxyanthocyanidins to the key targets.

## 3. Results

### 3.1. Structural Identification and Mass Spectrometry Behavior

The typical structures of new fermented deoxyanthocyanidins were identified and confirmed by NMR analysis and chemical synthesis [17], and 3D optimized geometrical structures of the typical deoxyanthocyanidins are shown in Figure S1. Although their corresponding mass spectrometry behaviors were discussed in detail before [17], the MS fragmented mechanisms of vinylphenol adducts of deoxyanthocyanidins can be elucidated here by quantum chemistry studies. Tandem mass spectrum of compound 7 (*m/z* 375.1222, vinylphenol-deoxyanthocyanidin) in Figure S2A showed that the fragmented ion of *m/z* 281.0805 in protic media corresponded to a molecular formula of C_17_H_13_O_4_^+^ and the loss of phenol. In deuterated methanol/water, deuterated compound 7 gave a precursor ion of *m/z* 379.1477 corresponding to a molecular formula of C_23_H_15_D_4_O_5_^+^ and the replacement of four protons with deuterium (Table 1). However, there were two deuterated fragmented ions of *m/z* 283.0931 (C_17_H_11_D_2_O_4_^+^) and *m/z* 284.0993 (C_17_H_10_D_3_O_4_^+^) in Figure S2B, which suggested that there were some potential fragmentation pathways on the loss of phenol from vinylphenol-deoxyanthocyanidin adducts in tandem MS.

**Figure 2:**
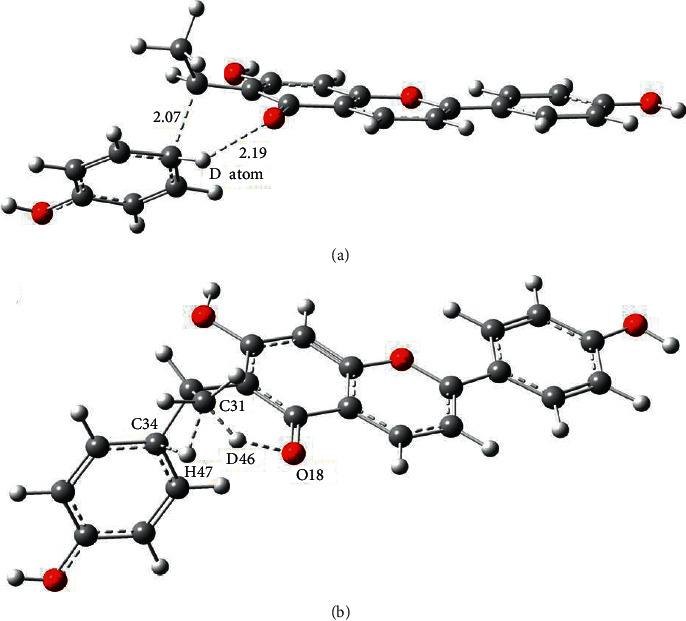
The transitional geometrical configurations of compound 7 by DFT calculations (Å). (a) The structure of TS1; (b) the structure of TS2.

**Figure 3:**
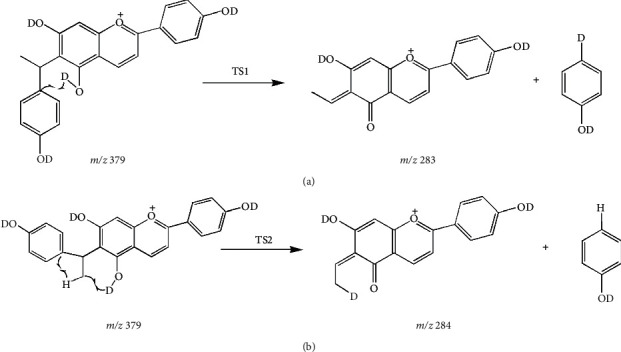
Proposed fragmentation pathways for deuterated fragmented ions of compound 7. (a) The pathway for *m/z* 283 *via* TS1; (b) the pathway for *m/z* 284 *via* TS2.

In order to illustrate the two deuterated fragment ions produced by deuterated compound 7 in Figure S2B, we discussed the possible transition states and corresponding energy barriers in the potential fragmentation pathways and verified the structures of transition states through IRC tests. For the deuterated fragment ion of *m/z* 283.0931 (C_17_H_11_D_2_O_4_^+^), the structure of transition state 1 (TS1) is shown in Figure 2(a), referring to the transfer of a deuterium atom to the leaving phenol *via* a six-membered ring transition (File S1). According to the IRC test, one side of the fragmentation pathway was the compound 7, and the other side was the dissociation products (File S2). As for the generation process of the deuterated product ion *m/z* 284.0993 (C_17_H_10_D_3_O_4_^+^), the potential fragmentation pathway involved dihydrogen cooperative transfer reactions in TS2 of Figure 2(b) (D46 from O18 to C31 and H47 to the leaving phenol group at C34) (File S3). IRC test (File S4) also proved that TS2 could connect with compound 7 and the dissociated product ion which contained three deuterium atoms in the ionic formula of C_17_H_10_D_3_O_4_^+^. The comparison of Gibbs free energy displayed that the energy of TS1 was approximately 47.45 kcal/mol lower than that of TS2, so TS1 was relatively easily to take place. The new proposed fragmentation pathways of deuterated compound 7 are shown in Figure 3.

### 3.2. Target Identification and Compound–Disease Regulatory Networks

Drugs can bind to multiple targets, and molecular targets can participate in multiple processes [31]. Thus, identifying the targets of deoxyanthocyanins is necessary to understand the molecular mechanism of these molecules in UC therapy. In this work, the SwissTargetPrediction database was used to predict the targets of these compounds and identify the top 100 potential human protein targets of each deoxyanthocyanin. A total of 529 UC-related targets were obtained through the GeneCard and Drug Bank databases (Table S1).

The deoxyanthocyanin–UC regulatory networks presented in Figure S3 contain 146 and 57 intersecting genes of natural and fermented deoxyanthocyanins, respectively. For natural deoxyanthocyanins, compound 5 connected with the largest number of related genes. For fermented deoxyanthocyanins, compound 7 (vinylphenol-deoxyanthocyanidin) is connected with the greatest number of related target genes. These results indicate that fermented deoxyanthocyanins may be more efficacious than natural compounds.

### 3.3. Gene Ontology Function and KEGG Enrichment Analyses

The functions of the target genes were determined by Gene Ontology (GO) analysis using the *R* package. The gene functions were divided into three categories, namely, biological process (BP), cellular component (CC), and molecular function (MF). The first seven enrichment results of GO analysis were visualized as bar graphs. For natural deoxyanthocyanins, BP is mainly involved in cellular response to oxidative stress, response to reactive oxygen species, and cellular response to reactive oxygen species; CC is mainly related to vesicle lumen, ficolin-1-rich granule lumen, and peroxisome; MF is mainly involved in heme binding, tetrapyrrole binding, and oxidoreductase activity, acting on paired donors, with incorporation or reduction of molecular oxygen (Figure S4A). For fermented deoxyanthocyanins, BP mainly involves aspects of response to oxidative stress, cellular response to reactive oxygen species, and reactive oxygen species metabolic process; CC is mainly related to membrane raft, membrane microdomain, and membrane region; MF mainly involves aspects of protein tyrosine kinase activity, heme binding, and *G* protein-coupled chemo attractant receptor activity (Figure S4B).

**Figure 4:**
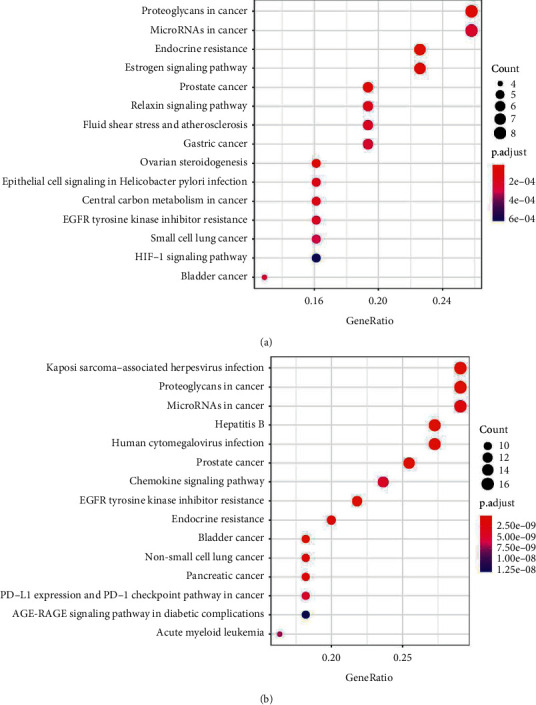
KEGG bubble. The horizontal axis of the KEGG bubble diagram represents the gene proportion enriched in each entry, and the vertical axis shows the enrichment degree according to the corrected *p* value.

The potential pharmacological mechanisms of deoxyanthocyanins against UC were revealed by KEGG enrichment analysis. According to the results of KEGG enrichment, the mechanisms of natural deoxyanthocyanins in the alleviation of UC mainly focus on proteoglycans in cancer, microRNAs in cancer, endocrine resistance, estrogen signaling pathway, prostate cancer, etc (Figure 4(a)); the mechanisms of fermented deoxyanthocyanins are mainly concentrated in Kaposi sarcoma-associated herpesvirus infection, proteoglycans in cancer, microRNAs in cancer, hepatitis B, human cytomegalovirus infection, etc (Figure 4(b)). The molecular functions and biological processes obtained were closely associated with the occurrence and development of UC. This finding indicates that deoxyanthocyanins can alleviate UC through a variety of targets and pathways.

### 3.4. Integration of Protein–Protein Interaction Networks

**Figure 5:**
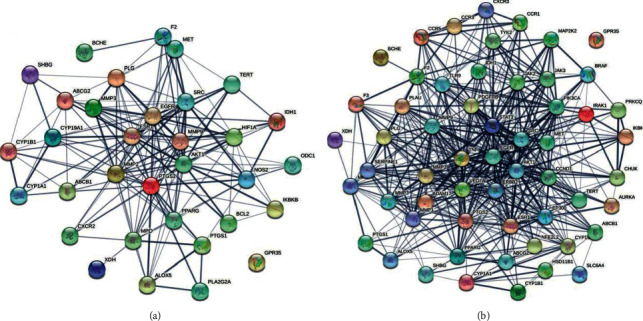
Topological analysis of the protein-protein interaction network. (a) Deoxyanthocyanidins before fermentation; (b) fermented deoxyanthocyanidins.

Following the prediction of target gene interactions using the STRING database, PPI networks were constructed to identify the most important proteins and biological molecules responsible for the effect of deoxyanthocyanins against UC. For natural deoxyanthocyanins, a total of 33 nodes and 160 edges were modulated by the PPI network (Figure 5(a)), and the 10 central nodes were identified as PTGS2, AKT1, ESR1, EGFR, SRC, MMP9, MMP2, HIF1A, MPO, and PPARG. Similarly, the PPI network of fermented deoxyanthocyanins included 57 nodes and 495 edges (Figure 5(b)), and the top 10 key genes were identified as TNF, VEGFA, STAT3, EGFR, PTGS2, CCND1, MMP9, ESR1, ERBB2, and JAK2. The hub genes common to these two networks (i.e., PTGS2, ESR1, and EGFR) were selected for further molecular docking studies to compare the effects of deoxyanthocyanins against UC before and after fermentation.

### 3.5. Comparison of Hub Targets by Molecular Docking

Three typical deoxyanthocyanins were selected as molecular docking ligands to compare the effects of natural and fermented deoxyanthocyanins against UC. The optimized structures of apigeninidin, compound 7, and compound 9 were separately uploaded to CB-Dock for docking analysis with PTGS2, ESR1, and EGFR as targets. Vina scores lower than 0 indicate that the target protein and ligand can bind spontaneously, and lower docking scores reflect more stable conformations. The docking scores of the first five conformations in each docking result are shown in Table S2, and conformations with the lowest docking scores are displayed in Table 2. The Vina scores obtained revealed strong interactions between different types of deoxyanthocyanins and the target proteins. Moreover, fermented deoxyanthocyanins showed lower scores compared with natural deoxyanthocyanins, which suggest that the former may be better able to alleviate UC than the latter. Detailed diagrams of the docking results of target proteins and ligands are shown in Figure S5.

## 4. Discussion

In the present study, a comprehensive network pharmacological method based on novel fermented vinylphenol/pyrano-deoxyanthocyanins and a disease database search was used to identify the most useful molecular targets and mechanisms of deoxyanthocyanins to alleviate UC. The novel fermented deoxyanthocyanidins were created *via* the reaction of natural deoxyanthocyanins (i.e., apigeninidin and methoxyapigeninidin) with the decarboxylation products of phenolic acids, such as hydroxycinnamic acid, coumaric acid, caffeic acid, and ferulic acid [32]. The use of decarboxylase-positive or -negative lactobacilli during fermentation indicated that the formation of fermented deoxyanthocyanidins depends on the bacterial decarboxylation of phenolic acids [17]. In the present study, the fragmentation pathways and transition states of deuterated vinylphenol-deoxyanthocyanins were investigated by DFT calculations of the results of MS/MS. Because the permanent positive charge of anthocyanin is located on the oxygen atom of the benzopyrylium, the number of phenolic hydroxyl groups in the structure of anthocyanin metabolites can be determined by the number of deuterium atoms in MS1 of the HDX-MS experiments [33]. In MS2, the possible structures of unknown anthocyanin metabolites may be deduced from HDX-MS experiments by comparing the fragmented mechanisms of known anthocyanin analogs [34]. Quantum chemistry can facilitate the interpretation, prediction, and analysis of MS data, particularly those obtained by MS/MS [35,36]. Using the tandem mass spectra of nondeuterated and deuterated vinylphenol-deoxyanthocyanins, we proposed two potential fragmentation pathways and calculated the optimized configurations, vibrational characteristics, IRCs, and relative energies of the transition states of these molecules using the DFT programme of Gaussian software. The results obtained from these analyses can support the results of previous studies and provide fundamental theories for further research on similar types of anthocyanins.

Comprehensive and user-friendly information on all annotated and predicted human genes is provided by GeneCards, which also integrates genomic, transcriptomic, proteomic, and clinical information related to genes [37]. DrugBank, which combines bioinformatics and cheminformatics, is the most reliable database containing detailed information for drugs, drug actions, and drug targets currently available [38]. In total, 529 genes related to UC were determined in the present study. We then used the SwissTargetPrediction database and Cytoscape software to intersect the targets of natural and fermented deoxyanthocyanins and obtain ‘component-target-disease' mappings. GO and KEGG pathway enrichment analysis were subsequently conducted using the *R* package. The results are given as follows: (1) for BP, the targets of natural and fermented deoxyanthocyanins had significant enrichment in response to oxidative stress, cellular response to reactive oxygen species, reactive oxygen species metabolic process, response to reactive oxygen species, and response to oxygen levels; (2) for CC, both of the targets were significantly enriched in membrane raft, membrane microdomain, vesicle lumen, and basal plasma membrane; (3) for MF, both of the targets were enriched in heme binding, tetrapyrrole binding, serine-type endopeptidase activity, and serine-type peptidase activity. For KEGG pathway analysis, both of the targets were particularly enriched in proteoglycans in cancer, microRNAs in cancer, prostate cancer, EGFR tyrosine kinase inhibitor resistance, and endocrine resistance (*P* < 0.05).

The PPI target networks of natural and fermented deoxyanthocyanins were separately constructed *via* the STRING online database. We comprehensively considered the top 10 targets of the two groups and selected PTGS2, ESR1, and EGFR as the hub targets of deoxyanthocyanins against UC. PTGS2 (or COX2; prostaglandin G/H synthase (2) plays a crucial role in the regulation of cellular responses to inflammation, which is a key factor in the pathogenesis of gut inflammation [39,40]. When luminal pathogens are stimulated via toll-like receptors, the expression and activity of PTGS2 are elevated to influence inflammatory processes through inhibition of NF-*κ*B, induction of PPAR-*γ*, and modification of the mucosal barrier function [41]. PTGS2 also possesses anti-inflammatory properties and acts as a proinflammatory cytokine [42]. ESR1 (estrogen receptor alpha, ER*α*) is a ligand-activated transcription factor that forms a homo- or heterodimer with ER*β* in the nucleus; this protein consists of several domains essential for hormone binding, DNA binding, and transcriptional activation [43]. In a clinical study, the ER gene showed age-related methylation during colorectal tumorigenesis, thereby suggesting that ER methylation in non-neoplastic epithelia could help predict the risk of UC-associated neoplasia in longstanding and extensive UC patients [44, 45]. EGFR (epidermal growth factor receptor) is a transmembrane glycoprotein that is mainly secreted by the submandibular and Brunner glands under physiological conditions [46, 47]. The biological functions of EGFR include cellular proliferation, differentiation, migration, and survival [47]. However, intestinal epithelial cells can also produce EGFR to cope with injury under pathological conditions [48]. Thus, EGFR plays an important role in the regulation of colonic epithelial biology and response to injury and inflammation [49].

Molecular docking studies are conducted to evaluate the binding orientation and affinity of small molecules to their targets [50]; this method was used in the present study to compare binding energies and action sites based on protein structures. According to our molecular docking results, fermented deoxyanthocyanins show potent anti-inflammatory and antioxidant effects and have a superior affinity toward their hub targets compared with natural deoxyanthocyanins. Fermented deoxyanthocyanins may have larger conjugate *π* systems and more phenolic hydroxyl groups than natural ones, and these characteristics may endow the former with enhanced anti-inflammatory abilities against UC [51, 52]. UC is an inflammatory disease associated with dysfunctions in intestinal immune recognition. The complex pathogenesis, lack of effective treatments, and common occurrence of various drug-related complications in UC has drawn attention to alternative treatments to prevent the progression of the disease [53]. For example, the use of symbiotic bacteria to regulate the intestinal microbiota and immune system to alleviate UC has been investigated [54]. The fermentation of carbohydrates, such as oligosaccharides, has been proven to have beneficial effects on gastrointestinal health, as well as the prevention or treatment of colorectal diseases [55]. In our study, we verified through network pharmacology that deoxyanthocyanins show significantly improved anti-inflammatory effects toward UC after fermentation. Because deoxyanthocyanins can only be fermented by specific probiotics, the use of these molecules to adjust the structure of the gut microbiota and resist UC may be feasible. Our research not only enriches the current understanding of UC-focused dietary therapy but also provides a candidate compound library for the discovery of new active compounds against the disease.

## 5. Conclusion

The purpose of this study is to analyze the mass spectrometric behavior of vinylphenol-deoxyanthocyanins by using DFT calculations and identify the molecular mechanisms of these molecules in alleviating UC *via* network pharmacology. We determined the potential targets and pathways of deoxyanthocyanins against UC responsible for their anti-inflammatory and antioxidative properties. We also screened out three hub targets (i.e., PTGS2, ESR1, and EGFR) and compared their binding energies with those of natural and fermented deoxyanthocyanins through the molecular docking method. Although the underlying mechanisms of deoxyanthocyanins toward their targets require further characterization *in vivo* and *in vitro*, *in silico* predictions clearly show that the effects of deoxyanthocyanins on alleviating UC are significantly enhanced following fermentation. For the first time, this work shows that HDX-MS is suitable for the analysis and identification of unknown deoxyanthocyanidin metabolites and the increase of pharmaceutical effects to alleviate UC after the metabolism of probiotics *via* network pharmacology.

## Figures and Tables

**Table 1:** The accurate mass data and elemental compositions of ions in Figure S2.

No.	Formula	Measured mass (m/*z*)	Theoretical mass (m/*z*)	Error (ppm)
1	C_23_H_19_O_5_^+^	375.1222	375.1227	1.33
2	C_17_H_13_O_4_^+^	281.0805	281.0808	1.07
3	C_23_H_15_D_4_O_5_^+^	379.1477	379.1478	0.26
4	C_17_H_11_D_2_O_4_^+^	283.0931	283.0934	1.06
5	C_17_H_10_D_3_O_4_^+^	284.0993	284.0997	1.41

**Table 2:** The lowest vina score for each docking.

Receptors	Apigeninidin	Compound 7	Compound 9
PTGS2	−9.2	−10.7	−10.9
ESR1	−8.7	−9.6	−8.5
EGFR	−8	−8.5	−9.6

## Data Availability

The data used to support the findings of this study are available from the corresponding author upon request.

## Abbreviations

UC: Ulcerative colitis
MS: Mass spectrometry
GO: Gene ontology
PPIs: Protein–protein interactions
STRING: Search Tool for the Retrieval of Interacting Genes
IBD: Inflammatory bowel disease
DSS: Dextran sodium sulfate
HDX-MS: Hydrogen deuterium exchange mass spectrometry
HRMS: High-resolution MS
DFT: Density functional theory
HCD: Higher energy collision-induced dissociation
NCE: Normalized collision energy
IRC: Intrinsic reaction coordinate
BP: Biological processes
CC: Cellular composition
MF: Molecular function
KEGG: Kyoto Encyclopedia of Genes and Genomes
PDB: Protein data bank
TS1: Transition state 1
EGFR: Epidermal growth factor receptor.

## Supplementary Materials

Supplementary Materials Table S1. UC-related targets from Genecard and Drugbank. Table S2. First five vina scores and cavity information of the docking simulation pose for deoxyanthocyanins and targeted proteins. File S1. The Gaussian file for the TS1 structure of compound 7. File S2. The Gaussian file for the IRC test of compound 7's TS1. File S3. The Gaussian file for the TS2 structure of compound 7. File S4. The Gaussian file for the IRC test of compound 7's TS2. Figure S1. The optimized geometrical structures by DFT calculations. (A) Apigeninidin; (B) compound 7 (vinylphenol-deoxyanthocyanidin); (C) compound 9 (pyrano-deoxyanthocyanidin). Figure S2. Tandem MS spectra of compound 7 (vinylphenol-deoxyanthocyanidin) by LTQ-Orbitrap MS. (A) Non-deuterated compound 7; (B) deuterated compound 7. Figure S3. Compound-disease regulatory networks about the targeted relationship between the deoxyanthocyanidins and the intersection genes. (A) Before fermentation; (B) after fermentation. Figure S4. GO enrichment analysis of deoxyanthocyanidins targets in treating UC. The horizontal axis of the BP, CC, and MF column represents the number of genes enriched in each item, and the color represents the enrichment significance based on the corrected *p* value. Figure S5. Schematic diagram of molecular docking. (A) PTGS2-apigeninidin; (B) PTGS2-compound 7; (C) PTGS2-compound 9; (D) ESR1-apigeninidin; (E) ESR1-compound 7; (F) ESR1-compound 9; (G) EGFR-apigeninidin; (H) EGFR-compound 7; (I) EGFR-compound 9.
