# Supplementary material for: Mass Spectrometric Behavior and Molecular Mechanisms of Fermented Deoxyanthocyanidins to Alleviate Ulcerative Colitis Based on Network Pharmacology
Source: Int J Anal Chem. 2022 Mar 21;2022:9293208. doi: 10.1155/2022/9293208 (PMC8960007; doi:10.1155/2022/9293208)
Supplement: Supplementary Materials — Table S1. UC-related targets from Genecard and Drugbank. Table S2. First five vina scores and cavity information of the docking simulation pose for deoxyanthocyanins and targeted proteins. File S1. The Gaussian file for the TS1 structure of compound 7. File S2. The Gaussian file for the IRC test of compound 7's TS1. File S3. The Gaussian file for the TS2 structure of compound 7. File S4. The Gaussian file for the IRC test of compound 7's TS2. Figure S1. The optimized geometrical structures by DFT calculations. (A) Apigeninidin; (B) compound 7 (vinylphenol-deoxyanthocyanidin); (C) compound 9 (pyrano-deoxyanthocyanidin). Figure S2. Tandem MS spectra of compound 7 (vinylphenol-deoxyanthocyanidin) by LTQ-Orbitrap MS. (A) Non-deuterated compound 7; (B) deuterated compound 7. Figure S3. Compound-disease regulatory networks about the targeted relationship between the deoxyanthocyanidins and the intersection genes. (A) Before fermentation; (B) after fermentation. Figure S4. GO enrichment analysis of deoxyanthocyanidins targets in treating UC. The horizontal axis of the BP, CC, and MF column represents the number of genes enriched in each item, and the color represents the enrichment significance based on the corrected p value. Figure S5. Schematic diagram of molecular docking. (A) PTGS2-apigeninidin; (B) PTGS2-compound 7; (C) PTGS2-compound 9; (D) ESR1-apigeninidin; (E) ESR1-compound 7; (F) ESR1-compound 9; (G) EGFR-apigeninidin; (H) EGFR-compound 7; (I) EGFR-compound 9. [file 9293208.f1.zip › 9293208.f1/Figure S3.pdf]

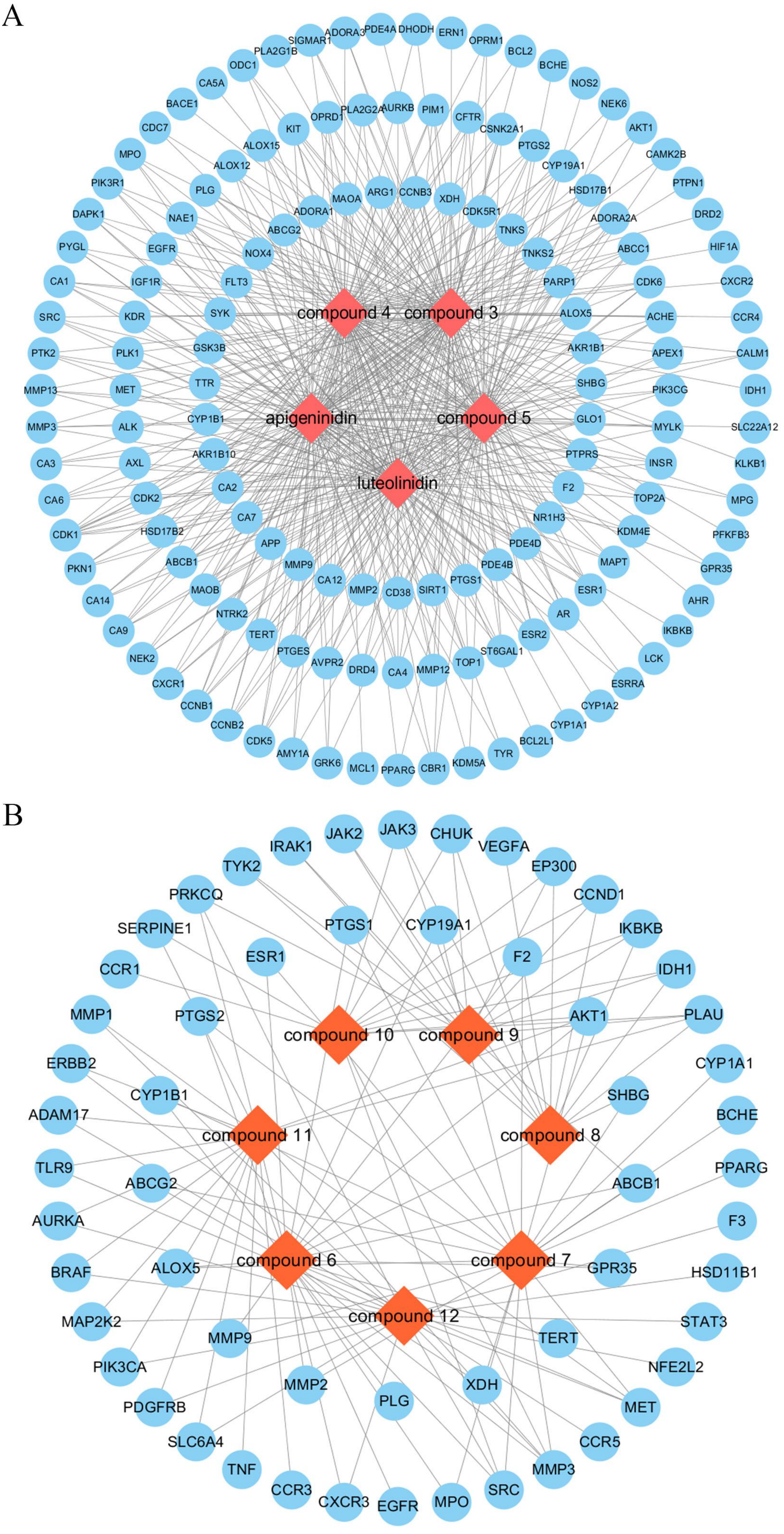
A
compound 4
compound 3
apigeninidin
compound 5
luteolinidin
SIGMAR1
ADORA3
PDE4A
DHODH
ERN1
OPRM1
BCL2
BCHE
NOS2
NEK6
AKT1
CAMK2B
PTPN1
DRD2
HIF1A
CXCR2
CCR4
CALM1
IDH1
SLC22A12
KLKB1
MPG
PFKFB3
GPR35
AHR
IKBKB
LCK
ESRRA
CYP1A2
CYP1A1
BCL2L1
TYR
KDM5A
CBR1
PPARG
MCL1
GRK6
AMY1A
CDK5
CCNB2
CCNB1
CXCR1
NEK2
CA9
CA14
PKN1
CDK1
CA6
CA3
MMP3
MMP13
PTK2
SRC
CA1
PYGL
DAPK1
PIK3R1
MPO
CDC7
BACE1
CA5A
ODC1
PLA2G1B
OPRD1
PLA2G2A
AURKB
PIM1
CFTR
CSNK2A1
PTGS2
CYP19A1
HSD17B1
ADORA2A
ABCC1
CDK6
ACHE
APEX1
PIK3CG
MYLK
INSR
TOP2A
KDM4E
MAPT
ESR1
AR
ESR2
ST6GAL1
TOP1
MMP12
CA4
DRD4
AVPR2
PTGES
TERT
NTRK2
MAOB
ABCB1
HSD17B2
CDK2
AXL
ALK
MET
PLK1
KDR
IGF1R
EGFR
NAE1
PLG
ALOX12
ALOX15
KIT
MAOA
ARG1
CCNB3
XDH
CDK5R1
TNKS
TNKS2
PARP1
ALOX5
AKR1B1
SHBG
GLO1
PTPRS
F2
NR1H3
PDE4D
PDE4B
PTGS1
SIRT1
CD38
MMP2
CA12
MMP9
APP
CA7
CA2
AKR1B10
CYP1B1
TTR
GSK3B
SYK
FLT3
NOX4
ABCG2
ADORA1
B
compound 10
compound 9
compound 11
compound 8
compound 6
compound 7
compound 12
JAK2
JAK3
CHUK
VEGFA
EP300
CCND1
IKBKB
IDH1
PLAU
CYP1A1
BCHE
PPARG
F3
HSD11B1
STAT3
NFE2L2
MET
CCR5
MMP3
SRC
MPO
EGFR
CXCR3
CCR3
TNF
SLC6A4
PDGFRB
PIK3CA
MAP2K2
BRAF
AURKA
TLR9
ADAM17
ERBB2
MMP1
CCR1
SERPINE1
PRKCQ
TYK2
IRAK1
PTGS1
CYP19A1
ESR1
F2
PTGS2
AKT1
CYP1B1
SHBG
ABCG2
ABCB1
ALOX5
GPR35
MMP9
TERT
MMP2
XDH
PLG
