# Supplementary material for: Mass Spectrometric Behavior and Molecular Mechanisms of Fermented Deoxyanthocyanidins to Alleviate Ulcerative Colitis Based on Network Pharmacology
Source: Int J Anal Chem. 2022 Mar 21;2022:9293208. doi: 10.1155/2022/9293208 (PMC8960007; doi:10.1155/2022/9293208)
Supplement: Supplementary Materials — Table S1. UC-related targets from Genecard and Drugbank. Table S2. First five vina scores and cavity information of the docking simulation pose for deoxyanthocyanins and targeted proteins. File S1. The Gaussian file for the TS1 structure of compound 7. File S2. The Gaussian file for the IRC test of compound 7's TS1. File S3. The Gaussian file for the TS2 structure of compound 7. File S4. The Gaussian file for the IRC test of compound 7's TS2. Figure S1. The optimized geometrical structures by DFT calculations. (A) Apigeninidin; (B) compound 7 (vinylphenol-deoxyanthocyanidin); (C) compound 9 (pyrano-deoxyanthocyanidin). Figure S2. Tandem MS spectra of compound 7 (vinylphenol-deoxyanthocyanidin) by LTQ-Orbitrap MS. (A) Non-deuterated compound 7; (B) deuterated compound 7. Figure S3. Compound-disease regulatory networks about the targeted relationship between the deoxyanthocyanidins and the intersection genes. (A) Before fermentation; (B) after fermentation. Figure S4. GO enrichment analysis of deoxyanthocyanidins targets in treating UC. The horizontal axis of the BP, CC, and MF column represents the number of genes enriched in each item, and the color represents the enrichment significance based on the corrected p value. Figure S5. Schematic diagram of molecular docking. (A) PTGS2-apigeninidin; (B) PTGS2-compound 7; (C) PTGS2-compound 9; (D) ESR1-apigeninidin; (E) ESR1-compound 7; (F) ESR1-compound 9; (G) EGFR-apigeninidin; (H) EGFR-compound 7; (I) EGFR-compound 9. [file 9293208.f1.zip › 9293208.f1/Table S2.pdf]

Target ID	Apigeninidin								compound7								compound9							
	Vina score	Cavity size	Center			Size			Vina score	Cavity size	Center			Size			Vina score	Cavity size	Center			Size		
			x	y	z	x	y	z			x	y	z	x	y	z			x	y	z	x	y	z
PTGS2 (PDB:5F19)	-9.2	5179	12	53	17	33	21	29	-10.7	16615	23	39	39	35	35	35	-10.9	16615	23	39	39	35	35	35
	-9.1	4392	14	49	65	21	30	29	-10.7	5179	12	53	17	33	24	24	-10.9	3279	28	35	39	24	24	24
	-8.3	16615	23	39	39	35	35	35	-10.7	3279	28	35	39	24	24	24	-10.6	4392	14	49	65	24	30	24
	-8.3	3279	28	35	39	29	29	21	-9.9	4392	14	49	65	24	30	24	-10.3	5179	12	53	17	33	24	24
	-8.2	1247	32	38	18	21	21	33	-8.7	1247	32	38	18	24	24	33	-8.9	1247	32	38	18	24	24	33
ESR1 (PDB:3OS8)	-8.7	8546	16	19	-62	35	29	29	-9.6	2441	16	37	-67	35	24	24	-8.5	2611	4	26	-45	24	32	24
	-8.6	2338	29	6	-61	21	21	21	-8.6	2611	4	26	-45	24	32	24	-8	2441	16	37	-67	35	24	24
	-8.5	2441	16	37	-67	35	21	21	-7.6	8546	16	19	-62	35	24	24	-7.8	3064	23	29	-29	30	24	24
	-7.5	2611	4	26	-45	21	32	21	-7.2	3064	23	29	-29	30	24	24	-7.7	8546	16	19	-62	35	24	24
	-6.8	3064	23	29	-29	30	21	29	-4.9	2338	29	6	-61	24	24	24	-3.2	2338	29	6	-61	24	24	24
EGFR (PDB:6S9B)	-8	5296	-54	30	-1	35	35	21	-8.5	5296	-54	30	-1	35	35	24	-9.6	5296	-54	30	-1	35	35	24
	-7	133	-79	15	-19	21	21	21	-7.1	266	-63	31	6	24	24	24	-7.3	125	-61	22	8	24	24	24
	-7	125	-61	22	8	21	21	21	-6.8	218	-72	32	-11	24	24	24	-7.1	266	-63	31	6	24	24	24
	-6.3	218	-72	32	-11	21	21	21	-6.8	133	-79	15	-19	24	24	24	-7.1	133	-79	15	-19	24	24	24
	-6.1	266	-63	31	6	21	21	21	-6.7	125	-61	22	8	24	24	24	-6.9	218	-72	32	-11	24	24	24
