# Supplementary figures and images for: Mass Spectrometric Behavior and Molecular Mechanisms of Fermented Deoxyanthocyanidins to Alleviate Ulcerative Colitis Based on Network Pharmacology
Source: Int J Anal Chem. 2022 Mar 21;2022:9293208. doi: 10.1155/2022/9293208 (PMC8960007; doi:10.1155/2022/9293208)

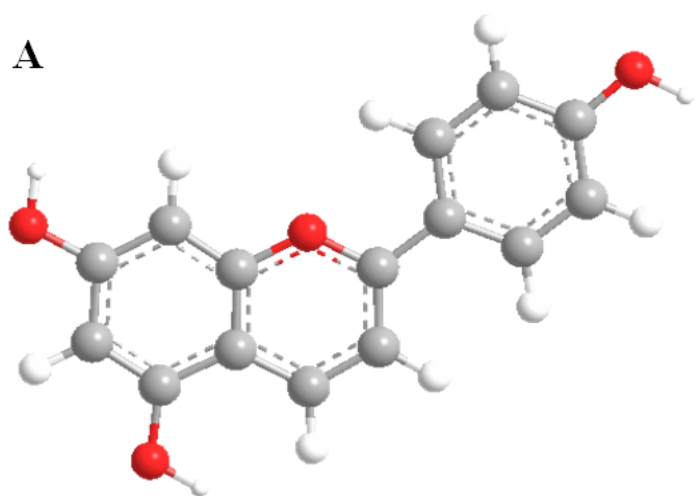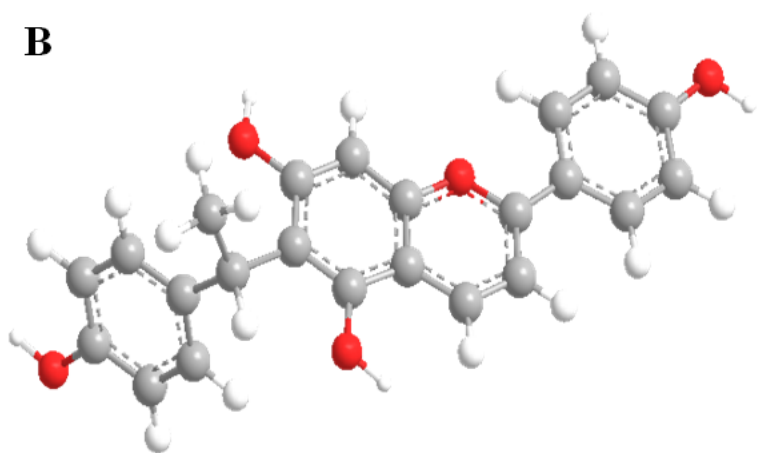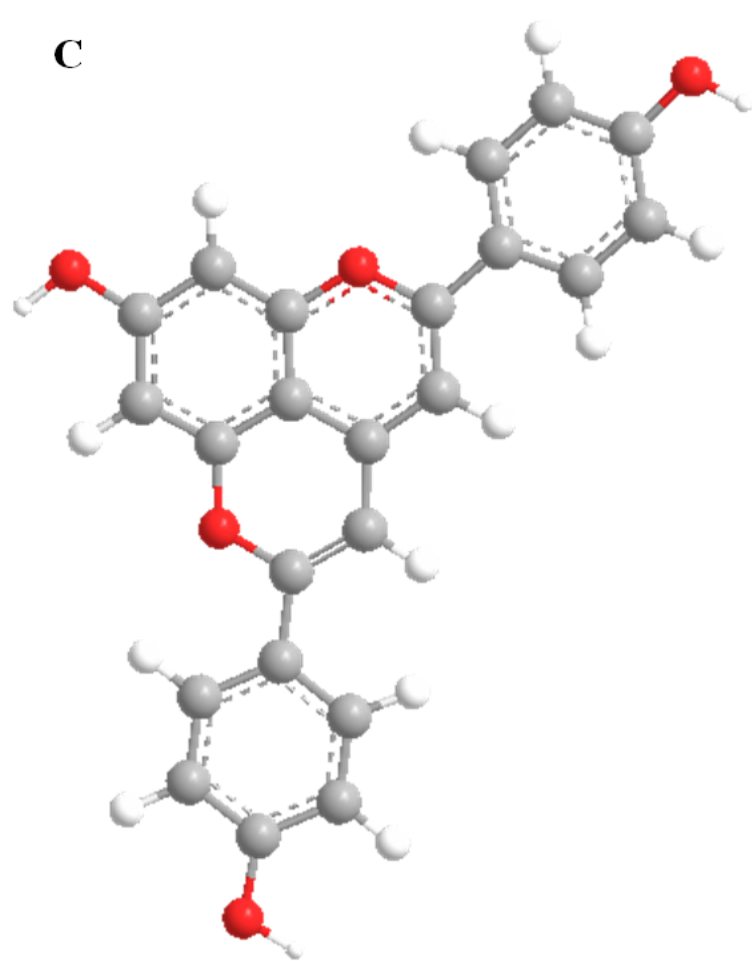

Supplement: Supplementary Materials — Table S1. UC-related targets from Genecard and Drugbank. Table S2. First five vina scores and cavity information of the docking simulation pose for deoxyanthocyanins and targeted proteins. File S1. The Gaussian file for the TS1 structure of compound 7. File S2. The Gaussian file for the IRC test of compound 7's TS1. File S3. The Gaussian file for the TS2 structure of compound 7. File S4. The Gaussian file for the IRC test of compound 7's TS2. Figure S1. The optimized geometrical structures by DFT calculations. (A) Apigeninidin; (B) compound 7 (vinylphenol-deoxyanthocyanidin); (C) compound 9 (pyrano-deoxyanthocyanidin). Figure S2. Tandem MS spectra of compound 7 (vinylphenol-deoxyanthocyanidin) by LTQ-Orbitrap MS. (A) Non-deuterated compound 7; (B) deuterated compound 7. Figure S3. Compound-disease regulatory networks about the targeted relationship between the deoxyanthocyanidins and the intersection genes. (A) Before fermentation; (B) after fermentation. Figure S4. GO enrichment analysis of deoxyanthocyanidins targets in treating UC. The horizontal axis of the BP, CC, and MF column represents the number of genes enriched in each item, and the color represents the enrichment significance based on the corrected p value. Figure S5. Schematic diagram of molecular docking. (A) PTGS2-apigeninidin; (B) PTGS2-compound 7; (C) PTGS2-compound 9; (D) ESR1-apigeninidin; (E) ESR1-compound 7; (F) ESR1-compound 9; (G) EGFR-apigeninidin; (H) EGFR-compound 7; (I) EGFR-compound 9. [file 9293208.f1.zip › 9293208.f1/Figure S1.pdf]

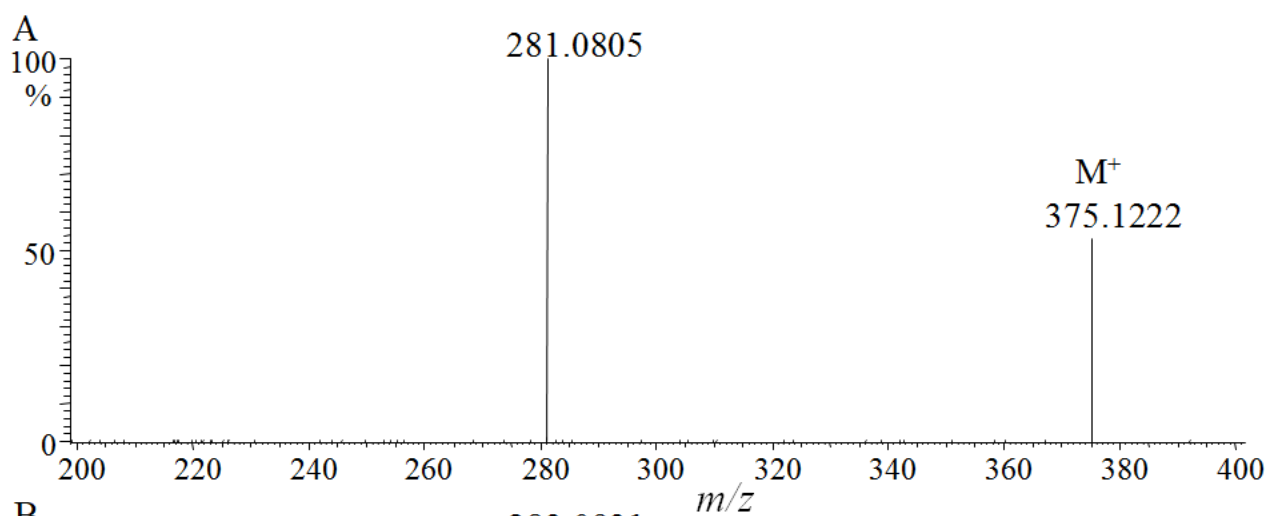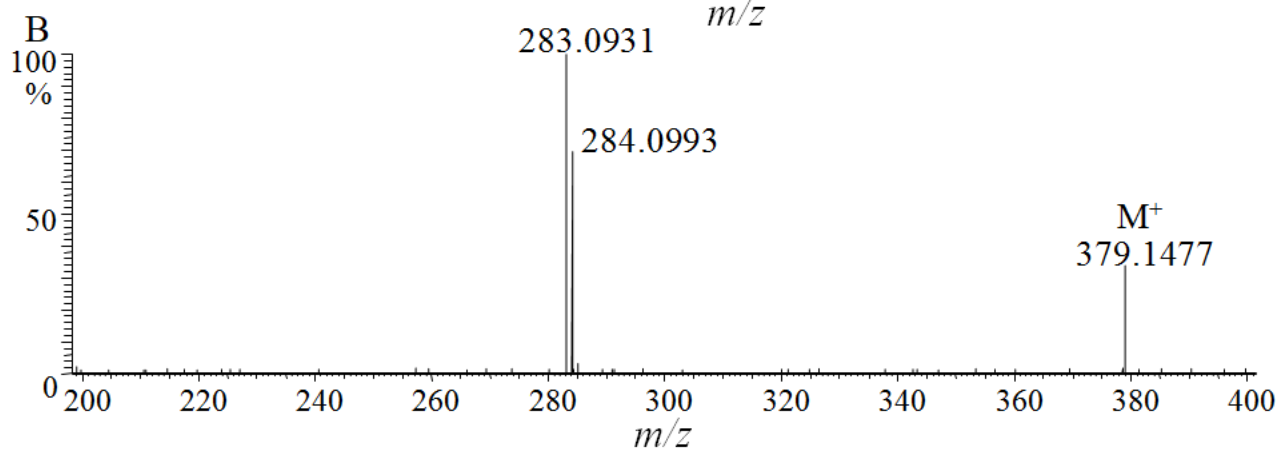

Supplement: Supplementary Materials — Table S1. UC-related targets from Genecard and Drugbank. Table S2. First five vina scores and cavity information of the docking simulation pose for deoxyanthocyanins and targeted proteins. File S1. The Gaussian file for the TS1 structure of compound 7. File S2. The Gaussian file for the IRC test of compound 7's TS1. File S3. The Gaussian file for the TS2 structure of compound 7. File S4. The Gaussian file for the IRC test of compound 7's TS2. Figure S1. The optimized geometrical structures by DFT calculations. (A) Apigeninidin; (B) compound 7 (vinylphenol-deoxyanthocyanidin); (C) compound 9 (pyrano-deoxyanthocyanidin). Figure S2. Tandem MS spectra of compound 7 (vinylphenol-deoxyanthocyanidin) by LTQ-Orbitrap MS. (A) Non-deuterated compound 7; (B) deuterated compound 7. Figure S3. Compound-disease regulatory networks about the targeted relationship between the deoxyanthocyanidins and the intersection genes. (A) Before fermentation; (B) after fermentation. Figure S4. GO enrichment analysis of deoxyanthocyanidins targets in treating UC. The horizontal axis of the BP, CC, and MF column represents the number of genes enriched in each item, and the color represents the enrichment significance based on the corrected p value. Figure S5. Schematic diagram of molecular docking. (A) PTGS2-apigeninidin; (B) PTGS2-compound 7; (C) PTGS2-compound 9; (D) ESR1-apigeninidin; (E) ESR1-compound 7; (F) ESR1-compound 9; (G) EGFR-apigeninidin; (H) EGFR-compound 7; (I) EGFR-compound 9. [file 9293208.f1.zip › 9293208.f1/Figure S2.pdf]

**A**

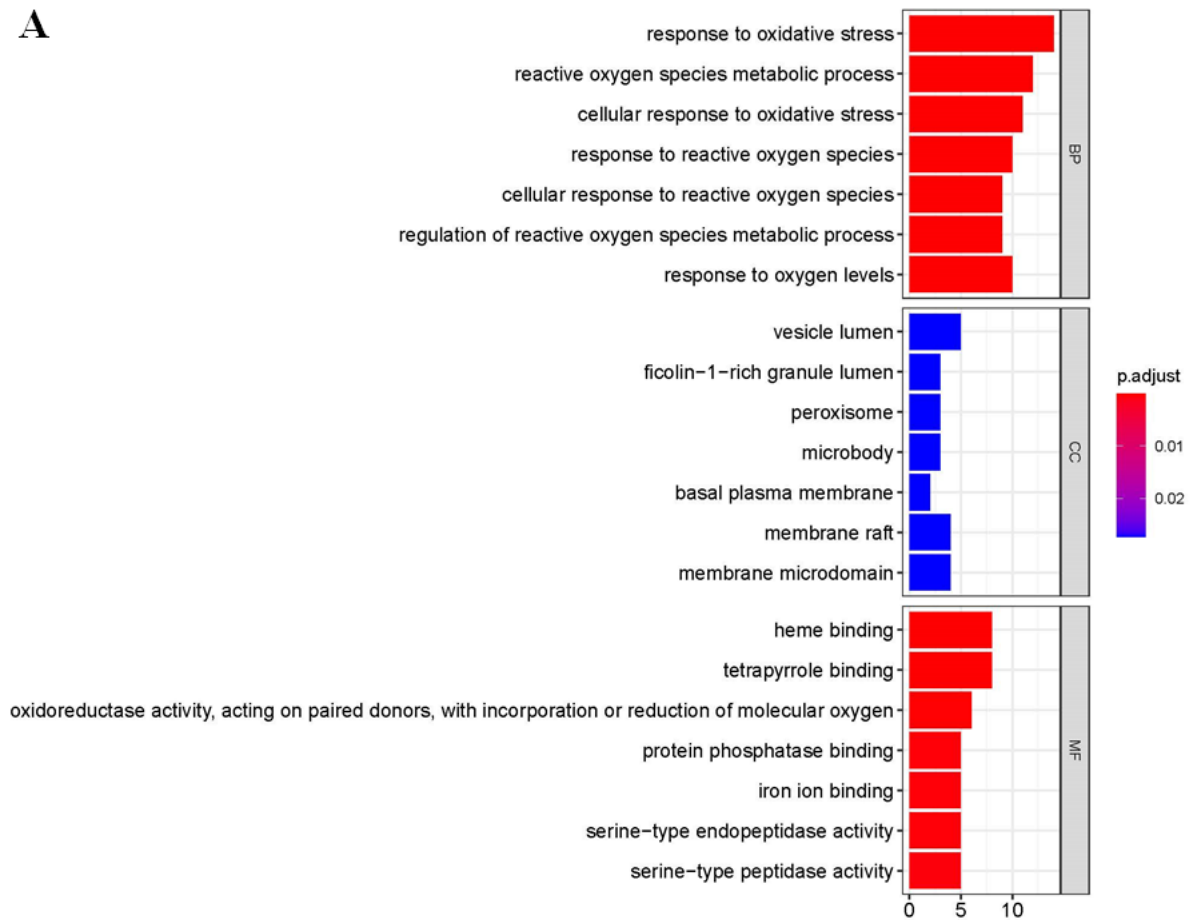

**B**

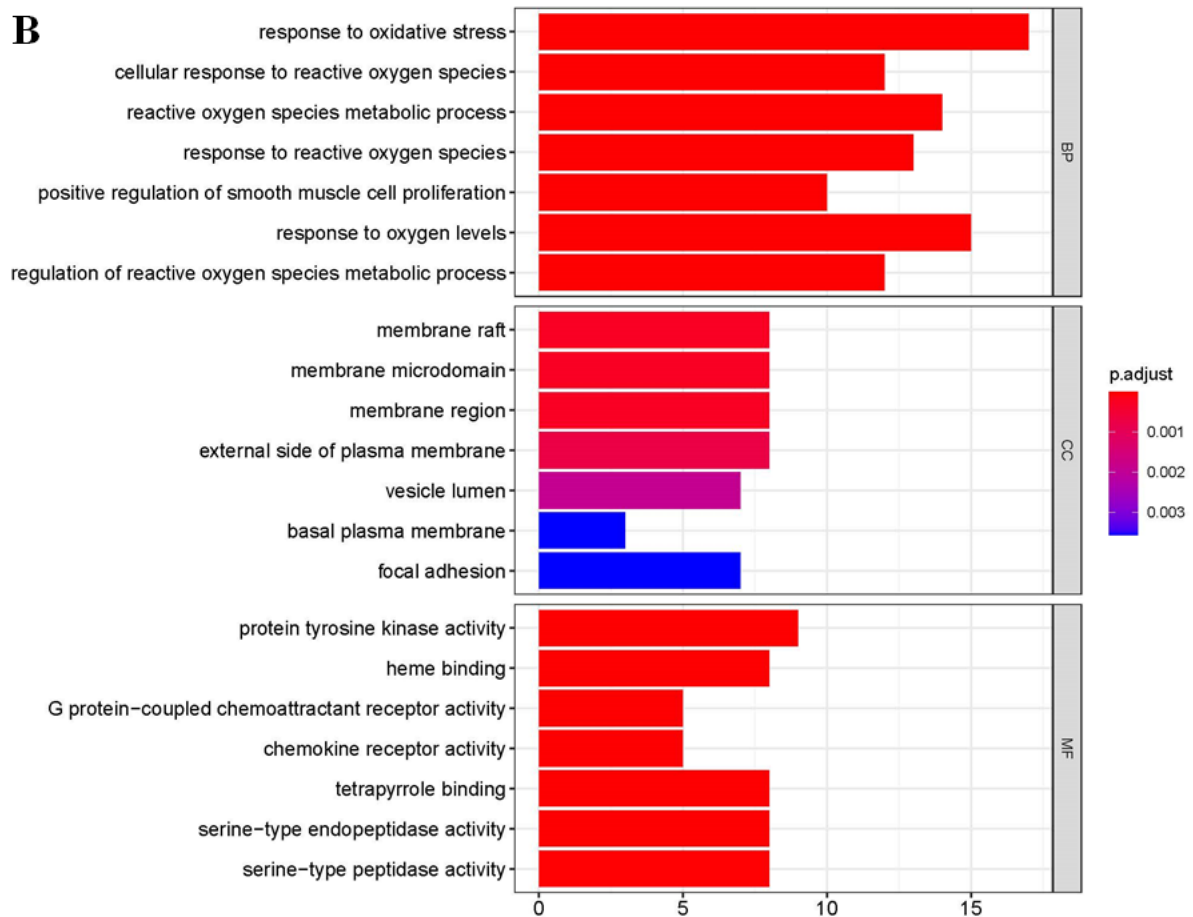

Supplement: Supplementary Materials — Table S1. UC-related targets from Genecard and Drugbank. Table S2. First five vina scores and cavity information of the docking simulation pose for deoxyanthocyanins and targeted proteins. File S1. The Gaussian file for the TS1 structure of compound 7. File S2. The Gaussian file for the IRC test of compound 7's TS1. File S3. The Gaussian file for the TS2 structure of compound 7. File S4. The Gaussian file for the IRC test of compound 7's TS2. Figure S1. The optimized geometrical structures by DFT calculations. (A) Apigeninidin; (B) compound 7 (vinylphenol-deoxyanthocyanidin); (C) compound 9 (pyrano-deoxyanthocyanidin). Figure S2. Tandem MS spectra of compound 7 (vinylphenol-deoxyanthocyanidin) by LTQ-Orbitrap MS. (A) Non-deuterated compound 7; (B) deuterated compound 7. Figure S3. Compound-disease regulatory networks about the targeted relationship between the deoxyanthocyanidins and the intersection genes. (A) Before fermentation; (B) after fermentation. Figure S4. GO enrichment analysis of deoxyanthocyanidins targets in treating UC. The horizontal axis of the BP, CC, and MF column represents the number of genes enriched in each item, and the color represents the enrichment significance based on the corrected p value. Figure S5. Schematic diagram of molecular docking. (A) PTGS2-apigeninidin; (B) PTGS2-compound 7; (C) PTGS2-compound 9; (D) ESR1-apigeninidin; (E) ESR1-compound 7; (F) ESR1-compound 9; (G) EGFR-apigeninidin; (H) EGFR-compound 7; (I) EGFR-compound 9. [file 9293208.f1.zip › 9293208.f1/Figure S4.pdf]

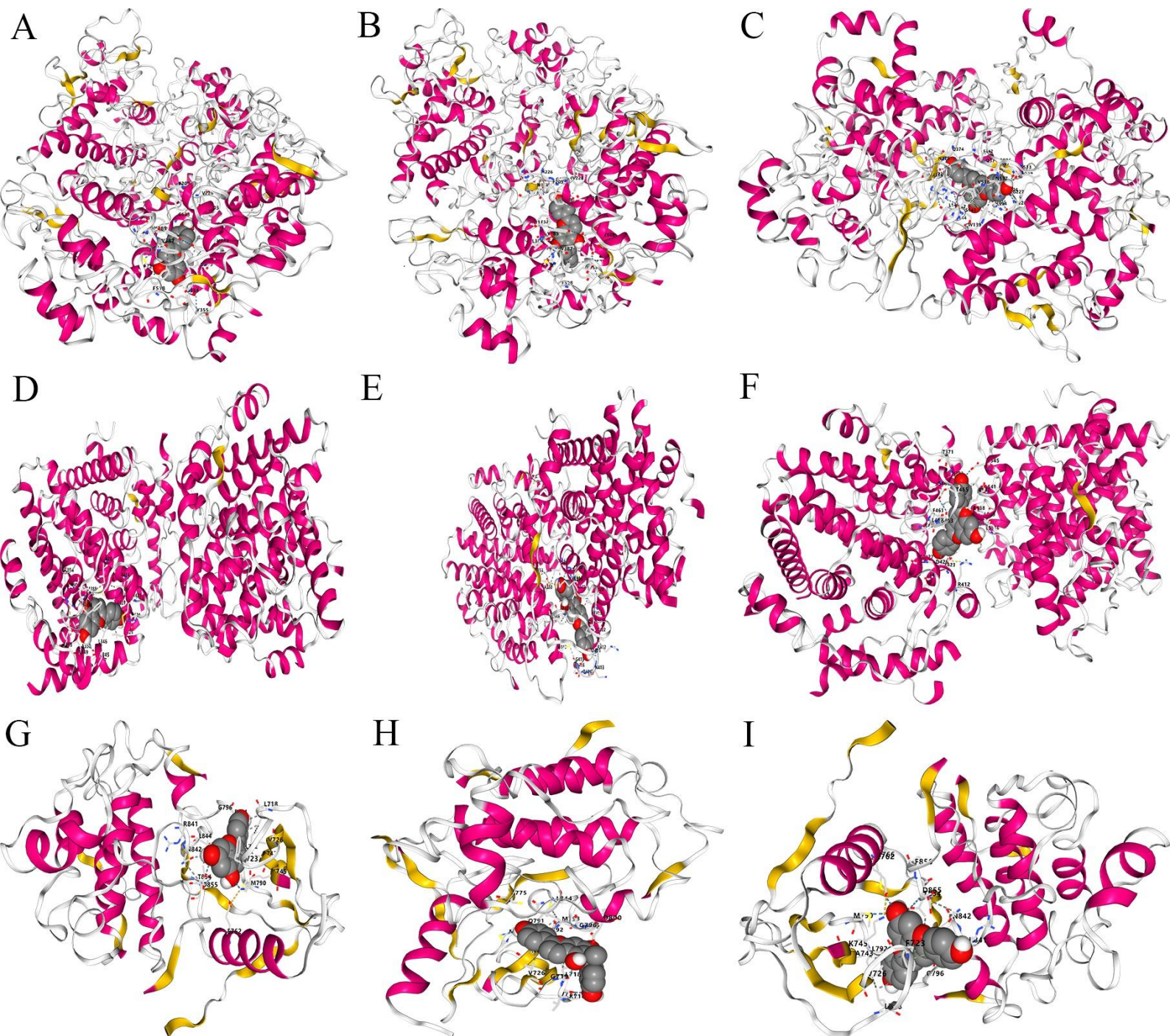

Supplement: Supplementary Materials — Table S1. UC-related targets from Genecard and Drugbank. Table S2. First five vina scores and cavity information of the docking simulation pose for deoxyanthocyanins and targeted proteins. File S1. The Gaussian file for the TS1 structure of compound 7. File S2. The Gaussian file for the IRC test of compound 7's TS1. File S3. The Gaussian file for the TS2 structure of compound 7. File S4. The Gaussian file for the IRC test of compound 7's TS2. Figure S1. The optimized geometrical structures by DFT calculations. (A) Apigeninidin; (B) compound 7 (vinylphenol-deoxyanthocyanidin); (C) compound 9 (pyrano-deoxyanthocyanidin). Figure S2. Tandem MS spectra of compound 7 (vinylphenol-deoxyanthocyanidin) by LTQ-Orbitrap MS. (A) Non-deuterated compound 7; (B) deuterated compound 7. Figure S3. Compound-disease regulatory networks about the targeted relationship between the deoxyanthocyanidins and the intersection genes. (A) Before fermentation; (B) after fermentation. Figure S4. GO enrichment analysis of deoxyanthocyanidins targets in treating UC. The horizontal axis of the BP, CC, and MF column represents the number of genes enriched in each item, and the color represents the enrichment significance based on the corrected p value. Figure S5. Schematic diagram of molecular docking. (A) PTGS2-apigeninidin; (B) PTGS2-compound 7; (C) PTGS2-compound 9; (D) ESR1-apigeninidin; (E) ESR1-compound 7; (F) ESR1-compound 9; (G) EGFR-apigeninidin; (H) EGFR-compound 7; (I) EGFR-compound 9. [file 9293208.f1.zip › 9293208.f1/Figure S5.pdf]
